# Gain-of-Function Screen for Genes That Affect *Drosophila* Muscle Pattern Formation 

**DOI:** 10.1371/journal.pgen.0010055

**Published:** 2005-10-28

**Authors:** Nicole Staudt, Andreas Molitor, Kalman Somogyi, Juan Mata, Silvia Curado, Karsten Eulenberg, Martin Meise, Thomas Siegmund, Thomas Häder, Andres Hilfiker, Günter Brönner, Anne Ephrussi, Pernille Rørth, Stephen M Cohen, Sonja Fellert, Ho-Ryun Chung, Olaf Piepenburg, Ulrich Schäfer, Herbert Jäckle, Gerd Vorbrüggen

**Affiliations:** 1 Max Planck Institut für biophysikalische Chemie, Göttingen, Germany; 2 DeveloGen, Göttingen, Germany; 3 Developmental Biology Unit, European Molecular Biology Laboratory, Heidelberg, Germany; North Carolina State University, United States of America

## Abstract

This article reports the production of an EP-element insertion library with more than 3,700 unique target sites within the *Drosophila melanogaster* genome and its use to systematically identify genes that affect embryonic muscle pattern formation. We designed a UAS/GAL4 system to drive GAL4-responsive expression of the EP-targeted genes in developing apodeme cells to which migrating myotubes finally attach and in an intrasegmental pattern of cells that serve myotubes as a migration substrate on their way towards the apodemes. The results suggest that misexpression of more than 1.5% of the *Drosophila* genes can interfere with proper myotube guidance and/or muscle attachment. In addition to factors already known to participate in these processes, we identified a number of enzymes that participate in the synthesis or modification of protein carbohydrate side chains and in Ubiquitin modifications and/or the Ubiquitin-dependent degradation of proteins, suggesting that these processes are relevant for muscle pattern formation.

## Synopsis

Muscle pattern formation during embryogenesis requires the activity of a distinct network of genes. In the model organism *Drosophila,* this process involves the determination of stem-cell-like muscle founder cells, their differentiation, and their attraction to tendon-like epidermal cells, termed apodemes, to which the muscles attach. In order to systematically identify genes involved in these processes, a collection of fruit fly strains was generated that can be used for the ectopic expression of more than 3,700 individual fruit fly genes in a spatiotemporally restricted manner. In order to address muscle pattern formation, the collection was used to express the genes in the developing apodemes and in a series of distinct epidermal cells that serve as migration substrate for developing muscles towards the apodemes. In addition to already known factors, some 60 novel gene activities were found to interfere under these circumstances with the formation of the muscle pattern. In addition to providing a most valuable tool for the *Drosophila* community of researchers, the results provide a framework for a detailed analysis of the gene network and insight into molecular mechanisms underlying embryonic muscle pattern formation.

## Introduction

Whole genome sequences of many animals are now known, including those of *Caenorhabditis elegans,* human, mouse, and *Drosophila melanogaster* (see for example [1–4]). The task now facing biologists is to discover the functions of the annotated genes within the genomes. For some organisms, such as *C. elegans,* it is possible to adopt a systematic approach to ablate gene function by, for example, the RNA interference technique [5,6]. For *Drosophila melanogaster,* a widespread analysis of gene function has been undertaken by systematic EMS mutagenesis and transposon tagging approaches using the P-element [7,8]. However, since two-thirds of genes in *D. melanogaster* and in *C. elegans* cause only subtle or even unscorable mutant phenotypes [9], a complementary approach was used. This approach is based on conditional overexpression of genes in order to generate gain-of-function phenotypes. It involves upstream activating sequences (UASs) of yeast provided through a recombinant transposon insertion, termed EP-element [10]. The inserted UAS can be used to transcriptionally activate an endogenous gene next to the insertion site by the transgene-dependent expression of the yeast transcription factor GAL4 under the control of a constitutively active promoter or spatiotemporally regulated enhancer elements [10,11].

Here we describe a newly generated EP-element library composed of more than 3,700 unique insertion sites and their location within the *D. melanogaster* genome. We employed this and previously constructed EP-element libraries for a systematic gain-of-function screen to identify gene activities that interfere with the proper development of the segmentally repeated muscle pattern. We designed a GAL4-driver to express endogenous genes in single epidermal cell rows, one anterior and one posterior to the embryonic *engrailed* expression domain [12], asking whether misexpression of genes in these locations alters the identity and/or the spatial cues of cells and thereby interferes with the genetically controlled migration and pathfinding ability of myotubes, as well as their anchoring properties (reviewed in [13]).

Muscle pattern formation is a stereotyped and segmentally repeated developmental process. Once muscle founder cells are born and determined, they grow by fusion with undetermined muscle cells. The resulting myotubes extend via the growth of cone-like tips along the inner surface of epidermal cells (reviewed in [13]), which serve as a migration substrate towards a distinct set of tendon-like segment border cells, termed apodemes, to which myotubes finally attach [13,14]. *stripe,* which encodes a zinc-finger-type transcription factor, is essential for apodeme cell formation at the segment borders [15]. In *stripe* mutants, myotubes soon fail to be properly guided, indicating that developing apodeme cells not only serve as attachment sites but also provide guiding cues for the migrating myotubes [15,16]. In addition, myotube guidance is also controlled by the myotube-expressed gene *grip* [17], by Slit/Robo signaling [18,19], and the attachment to apodemes involves the atypical receptor tyrosine kinase Derailed [20] as well as fibroblast growth factor signaling activity [21]. These and other results [13] have established that myotube guidance and attachment are controlled by interactions between epidermal and muscle cells and that interfering with their interactions causes scorable effects on the stereotyped muscle pattern. Here, we describe a systematic gain-of-function screen towards identifying gene activities that can interfere with the formation of the proper muscle pattern in the *D. melanogaster* embryo by using the specially designed UAS/GAL4 misexpression system.

## Results/Discussion

### Generation of an EP-Element Insertion Collection

We generated a novel EP-element collection for *D. melanogaster*. It contains single insertions bearing GAL4-dependent UASs of yeast at their ends [22,23]. Genes properly oriented with respect to the UAS sequences can therefore be conditionally expressed via transgene-derived GAL4 activity [10].

Of more than 13,800 individual EP-element lines initially generated, the insertion sites of more than 11,700 individual lines were determined by a combined PCR/sequencing approach [22]. Among these insertion sites, we identified a total of 3,707 unique EP-element insertion sites within the *D. melanogaster* genome. Their location and the orientation of the EP-elements are summarized in Tables S1 and S2. Using this collection, roughly a quarter of the *D. melanogaster* genes [2] can be activated by transgene-derived GAL4 activity that is driven by constitutively active promoters or cell-specific enhancer elements [11]. In addition, a sizable portion of the EP-elements are in reverse orientation with respect to genes (that is, there are no other annotated transcription units within a range of 10 kb of genomic DNA [2]), suggesting that the activity of these genes would likely be knocked down in response to transgene-derived GAL4 activation (see Tables S1 and S2).

### Generation of a GAL4-Driver Line Causing Epidermal Stripe Expression

**Figure 1:**
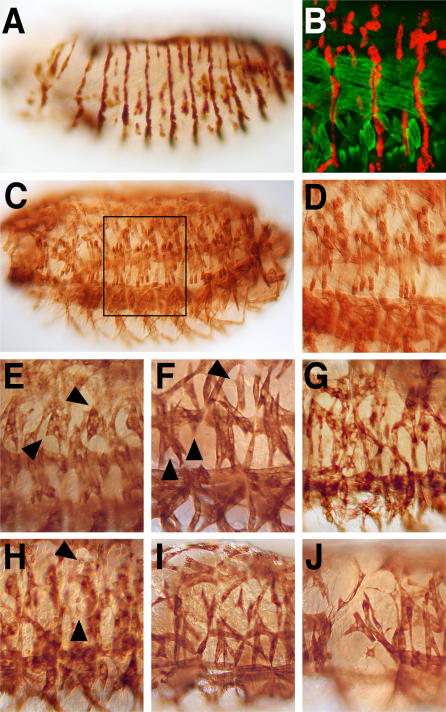
Expression Pattern of the *srmodGAL4*-Driver and Induced Muscle Pattern Defects in Response to EP Targeted Endogenous *D. melanogaster* Genes (A) Expression pattern of *srmodGAL4* driving a UAS-*lacZ* transgene in a stage 15 embryo (lateral view). Note the expression of β-Galactosidase in a segmentally repeated pattern of segment border cells, which appear as stripes, and in an array of partially interrupted intersegmental cell rows between them. (B) Enlarged lateral area of a stage 16 embryo stained with anti-MHC antibodies (green) to visualize the muscle pattern and anti-β-Galactosidase antibodies (red) to visualize the apodemes and intrasegmental epidermal cells that express the marker gene in response to *srmodGAL4* activity (red). (C and D) Muscle pattern (anti-MHC staining) of wild-type stage 16 embryos (C) and enlarged lateral area (D) outlined in (C). (E–J) Examples of muscle phenotypes (enlargements as in [D]) in response to *srmodGAL4*-driven misexpression of EP-targeted genes. Note that expression of CG9742 (HD10913) induces abnormal attachment of LT4 and LT5 muscles ([E], arrowheads), *adk1* (HD32155) affects the shape and attachment of all LT muscles ([F], arrowheads), *parg* (HD10914) causes an abnormal shape and attachment of all lateral muscles (G), and CG32436 (HD35012) impairs the fusion of myoblasts (see unfused MHC-stained myoblasts) ([H], arrowheads), whereas CG4963 (HD35059) (I) and CG31710 (EP2160) (J) affect determination, growth, and attachment of many muscles at the same time.

In order to perform a large-scale gain-of-function screen for gene activities that interfere with *D. melanogaster* muscle pattern formation during embryogenesis, we designed a GAL4-driver that allowed the misexpression of EP-targeted genes in presumptive apodeme precursors at the segment border and in an ectopic array of intrasegmental cell rows within the epidermis of the embryo. We made use of the *sr239* enhancer element of the *stripe* gene, which drives gene expression in a single cell row posterior to the *engrailed* expression domain. These cells correspond to dorsal and lateral apodeme precursor cells at a midstage of embryogenesis (stage 12) [12]. We fused this element with the GAL4 coding region to express UAS-targeted genes in a subset of apodeme cells. In addition, we used the *sr239*Δ*pan* enhancer [12], termed *srmod,* to drive GAL4 expression in apodeme cells and in a subset of epidermal cells anterior to the *engrailed* expression domain (Figure 1A and 1B) that serve as a migration substrate for myotubes. We expected these tools to facilitate the identification of genes whose activities interfere with processes such as myotube guidance or muscle attachment when expressed in response to one or both of the GAL4-drivers.

### Screening for Genes That Interfere with Muscle Pattern Formation

To activate misexpression of endogenous EP-targeted genes, we crossed females bearing the *srmodGAL4*-driver with male individuals from about 4,500 lines of the new (Table S1) and a previous EP-element collection [10] and asked whether the misexpression causes a lethal phenotype, knowing that impairing the stereotyped muscle pattern prevents the hatching of fully differentiated larvae from the egg shell [15,16]. In the next step, we examined whether muscle pattern defects can be observed after staining of the fully differentiated but unhatched embryos with anti–Myosin heavy chain (MHC) antibodies. To distinguish between interfering gene activities that were induced in the segment border apodeme cells and those that were derived from the intervening epidermal cells, we performed corresponding crosses using *sr*GAL4-bearing instead of *srmodGAL4*-bearing females.

We identified an initial set of 78 EP-element lines (1.7%) that caused a specific *srmodGAL4*-dependent muscle pattern phenotypes. To confirm that the observed phenotypes in the embryonic muscle pattern originated from misexpression of a given gene, we tested whether (i) the phenotype could be reverted by the precise excision of the EP-element, (ii) the potential target gene was expressed in a GAL4-dependent fashion (this was tested using in situ hybridization or antibody staining for product detection), (iii) the muscle pattern defects also occurred by over-expression of corresponding cDNA from UAS-dependent transgenes, or (iv) whether misexpression of the same transcription unit by a different EP-element insertion caused a similar phenotype.

The strength and penetrance of the misexpression muscle pattern phenotypes were variable (compare Figure 1C and 1D with 1E–1J). We found embryos in which only single muscle fibers were abnormally attached to apodemes (Figure 1E and 1F), embryos in which most muscles of the dorsal and lateral region of the embryo were abnormally shaped and attached to ectopic epidermal sites (Figure 1G), and embryos in which the early processes of myogenesis were aberrant, as concluded from impaired myoblast fusions (Figure 1H) and muscle misdetermination (Figure 1I and 1J). The different defects suggest that activities derived from the misexpressed genes can interfere with cell determination as well as guiding and targeting events during muscle pattern formation. In some cases, the defects observed were not restricted to dorsal and lateral muscles but also included muscles in the ventral region of the embryo, where few epidermal cells express *srmod*-dependent GAL4 activity (not shown).

### Gene Activities That Interfere with Muscle Growth and Attachment

**Figure 2:**
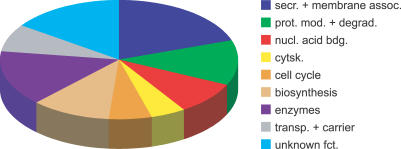
Schematic Representation of the Classification of the 66 Identified Candidate Genes into Functional Groups The affiliation of the genes products is indicated by the color and the size of the fragments represents the quantitative distribution. cytsk., cytoskeleton; nucl. acid bdg., nucleic acid binding; prot. mod. + degrade., protein modification or degradation; secr. + membrane assoc., secreted or membrane-associated factors; transp. + carrier, transporter or carrier; unknown fct., unknown function.

Of the initially identified 78 EP-element insertions, 66 GAL4-driven transcription units could be unambiguously identified to be the cause of the gain-of-function phenotypes (Table S3). Of those, ten transcription units were expressed in anti-sense orientation, implying that misexpression of transcripts in reverse orientation is likely to cause a knock-down phenotype. Analysis of the expression patterns of some of the anti-sense-tagged candidates indicated that the transcripts are expressed ubiquitously or accumulate at the segment border (see below). Thus, GAL4-driven misexpression may result in reduced gene activity. Fifty-six transcription units were in sense orientation, suggesting misexpression phenotypes in response to GAL4-drivers. Computer assisted analysis of the products of the targeted transcription units revealed that many of the candidates with known or predicted functions encode for membrane-associated or -secreted factors as well as for components known to be involved in protein modification and degradation (Figure 2).

#### Genes coding for membrane associated and secreted factors.

Thirteen genes encode proteins that contain diagnostic domains for membrane association or secretion. This group includes Tetraspanin Tsp42Ee (CG10106), one protein with three transmembrane domains (CG9030), and five factors with a single transmembrane domain that is typical for receptor-type proteins. This last group includes Toll*,* a receptor that participates in dorsoventral patterning of the embryo and innate immune response, and Syndecan (Sdc), a heparan sulfate proteoglycan (HSPG) that participates in Slit/Robo signaling [24,25]. Furthermore, membrane-associated factors were identified including CG33207/Pxb, which functions as an attenuator for *hedgehog* signaling [26], and the *polychaetoid* protein, a guanylate kinase at the adherens junctions that participates in JNK signaling [27].

The identification of a subset of transmembrane proteins in which two out of four proteins (Sdc and Toll) are already known to participate in muscle pattern formation [25,28] provides trust that other identified genes that code for membrane associated and secreted factors with unknown functions may also take part in the process. These uncharacterized factors include CG14052, CG6301, and CG17368, which encode small proteins containing an N-terminal signal peptide, implying that they represent secreted factors for which functions need to be established.

#### Protein modification and degradation.

A total of eight genes encode for factors involved in protein modification and degradation. Three genes encode components of the Ubiquitin pathway including *uba1* and *effete,* which encode E1 and E2 enzymes, respectively, as well as CG11033, which codes for an uncharacterized F-Box protein. F-Box proteins are required for target protein binding and for Ubiquitin transfer by the E2/E3 complex. Both *effete* and *uba1* have been shown to participate in neurogenesis [29,30]. *uba1* was initially found in a gain-of-function screen for genes involved in motor axon guidance [31].

Of the subset of proteins modifying enzymes, five play a role in modifying carbohydrate side chains of peptides. Of those, *sulfateless* encodes a heparan sulfate–glucosamine-N-sulfotransferase required for Decapentaplegic, Hedgehog, and fibroblast growth factor signaling [32]. The finding of a HSPG-modifying enzyme is consistent with the concurrent identification of the HSPG Sdc (see above), already known to affect muscle guidance [25,33]. We identified also a second sulfotransferase (CG32629/CG32632 fusion) and two genes that code for proteins that modify extracellular carbohydrates (CG31973 and *gnbp3*). The finding of several enzymes involved in carbohydrate side chain synthesis and modification suggests that they play a role not only in axon guidance [34] but also in muscle guidance and/or apodeme targeting. In addition, the identification of several Ubiquitin pathway components implicated in protein degradation suggests a role also of this process in muscle pattern formation.

#### Transcription factors and RNA binding proteins.

Only six potential or known DNA or RNA binding factors were identified. This result suggests that only a comparatively small number of transcription factors can interfere with the functional development of apodeme cells in a manner recently shown for the zinc finger protein encoded by *stripe* [15,16]. Interestingly, the identified transcription factors also include two zinc finger proteins, encoded by *schnurri* and *escargot (esg),* that have been shown to act in the formation of the tracheal system [35–37]. Esg is involved in Cadherin-mediated adhesion [37]. Thus, its misexpression may cause abnormal adhesion of muscles when *esg-*expressing epidermal cells are provided as a substrate. *schnurri* activity is required to properly mediate TGFβ signaling [35]. Its ectopic expression may therefore cause an improper signaling readout that impairs myotube outgrowth and/or muscle attachment.

#### Cytoskeleton factors.

Three genes code for known cytoskeleton binding proteins such as Katanin80, a WD40 domain microtubule binding protein of the Katanin complex involved in micotubule severing. In addition, we found *chickadee,* which was identified twice by independent EP-element insertions in this screen. *chickadee* protein is involved in Actin filament organization and contains a phosphatidylinositol-4,5-bisphophate binding motif. This motif is noteworthy with respect to *rdgB*β, which codes for a phosphatidylinositol transfer protein coupling phosphatidylinositol delivery and phosphatidylinositol-4,5-bisphophate synthesis relevant for cell–cell signaling processes (reviewed in [38]) and which was also identified in the screen.

#### Factors involved in cell cycle control and biosynthesis.

Seven factors involved in central steps of biosynthesis were identified. They include the ribosomal protein RpL18A and the polyadenylation binding protein Pabp2. In addition, cell cycle control genes such as the *D. melanogaster* CDC25 homolog *twine* and two *cycline* genes were found. Interference of overexpressed general biosynthesis factors and cell cycle control genes can be explained if they would alter proper epidermal cell differentiation, patterns of cell death, and/or patterns of cell divisions. In these cases, gene expression could impair processes required to maintain or generate properly differentiated epidermal cells that serve as substrate for the outgrowing myotubes or provide spatial cues relevant for this process.

### Gene Expression Patterns

We examined the expression pattern of a total of 46 of the identified genes. This criterion for validation of potential gene functions for muscle guidance and attachment control included whole mount in situ hybridization using anti-sense RNA probes prepared from respective cDNAs or genomic fragments covering parts of the identified candidates as well as information available from a *D. melanogaster* database [39].

**Figure 3:**
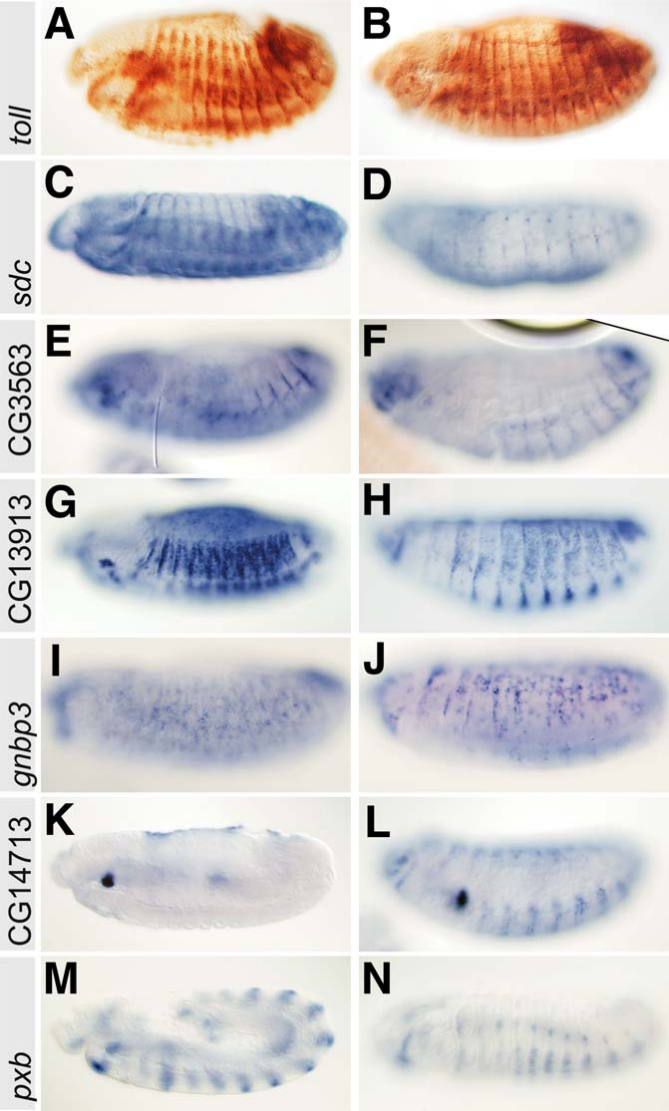
Expression Patterns of Genes that Cause a Gain-of-Function Muscle Phenotype Lateral views of embryos at stage 11 (M), stage 13 (A, C, E, G, I, K, and N), and stage 16 (B, D, F, H, J, and L) that were stained with transcript-specific anti-sense RNA probes or with anti-Toll antibodies (A and B). Note the expression of Toll (A and B) in segment border cells, *sdc* (LD08230) (C and D) in trachea, segment border cells, and the differentiated apodemes, CG3563 (LD15689) (E and F) in the apodeme precusor cells at the segment border, CG13913 (RE53394) (G and H) and CG5008/*gnbp3* (SD21560) (I and J) in a subset of apodeme precursors and cells of the epidermis, CG14713/14714 transcripts (AT17253) (K and L) in the dorsal and ventral epidermis around the segment border, and *pxb* (SD26190) (M and N) in intrasegmental epidermal stripes.

The majority of genes are expressed in patterns that could not be directly correlated with muscle pattern formation. However, most were either ubiquitously expressed or they were maternally contributed, and transcripts are present in eggs and during early embryogenesis. Yet, about one-third of the genes were expressed in spatiotemporal patterns in the epidermis during the stage when myotube migration takes place. Eight of these genes were expressed in the apodeme precursor cells of wild-type embryos, including seven of the 12 genes that encode cell surface proteins or secreted factors. Examples of the gene expression pattern are shown Figure 3.

The fraction of genes that are expressed in apodeme cells at the stage when they are targeted by the muscles includes *Toll, sdc,* CG3563, CG13913, and *gnbp3* (Figure 3A–3J). The expression patterns of Toll and Sdc have previously been described. Toll is expressed in a subset of the developing apodeme cells and participates in muscle pattern formation [28]. Sdc is expressed in the mesoderm, the tracheal system, the axons of the central nervous system and in the differentiated apodemes. In *sdc* mutants, muscles fail to respect the ventral midline as a migration border, cross the border, and subsequently attach to apodemes at the other side of the midline [25]. Other genes, such as CG14713 and *pxb*/AT17253, are expressed in the intrasegmental region of the epidermis that is crossed by the migrating myotubes (Figure 3K–3N).

It is noteworthy that the set of identified genes also includes genes that are normally expressed in cells of other tissues or organs whose development involves migratory processes of cells or groups of cells. These include the developing tracheal system, germ line precursor cells, the midgut, and the nervous system. Thus, although the expression patterns exclude a role for these genes during the normal process of muscle pattern formation, they could play a direct or indirect role in guiding migrating cells in regions of the wild-type embryo where they are normally expressed. Preliminary results with a gene specifically expressed in germ line precursor cells supports this proposal (G.V., unpublished data).

### Gene Activities Required for Muscle Pattern Formation

**Figure 4:**
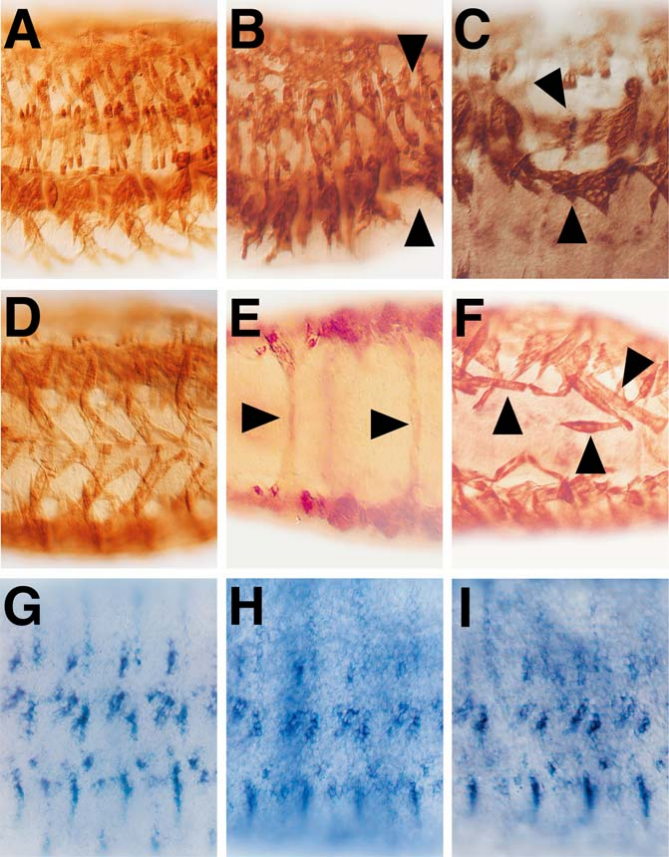
Muscle Pattern Defects in *esg* and *sdc* Mutants Muscle pattern of three segments of *oreR* (A, D, and G), *esg ^L2^* (B and H), and *sdc ^23^* mutant embryos (C, E, F, and I) after staining with anti-MHC antibodies or using a *delilah* transcript-specific anti-sense RNA probe (G–H). Lateral (A–C and G–I) and ventral views (D–F) of embryos at stage 14 (E) and stage 16 (A–D and F–I). *esg* mutant embryos show variable muscle pattern defects with muscles absent ([B], arrowheads). In *sdc* mutant embryos few muscles cross the ventral midline in a position dorsal to the central nervous system ([E], arrowheads), and they show disruptions of the pattern in the ventral region ([C], arrowheads). The typical “finger-type pattern” of the ventral muscles of wild-type embryos (D) is unordered in *sdc ^23^* mutant embryos, with ventral muscles aligning in parallel with the anterio-posterior axis, ignoring the segment border attachment ([F], arrowheads). Also shown is the pattern of epidermal muscle attachment sites (*delilah* marker gene expression) in wild-type (G), *esg* (H), and *sdc* (I) mutant embryos. Note that the pattern is unchanged in the mutants.

Muscle pattern phenotypes of *Toll*, *gut feeling (oda),* and *sulfateless* mutant embryos have already been described [28,32,40]. In order to test whether other genes that were identified in the gain-of-function screen also caused a loss-of-function phenotype, we examined the muscle pattern of loss-of-function mutants that were described in a context different from muscle pattern formation. Figure 4 shows two examples of the analysis, indicating that *esg* (compare Figure 4A with 4B) and *sdc* (compare Figure 4A and 4D with 4C, 4E, and 4F) loss-of-function mutant embryos develop variable muscle pattern defects that include the absence of lateral transverse muscles, loss of muscle fibers, and abnormally shaped muscles. Since *sdc* and *esg* are expressed in the epidermis, we used expression of *delilah,* a marker for the muscle attachment sites [41], to examine whether an altered pattern of attachment sites is a likely cause of the muscle pattern defects. No pattern defects were observed in *sdc* and *esg* mutants (Figure 4G–4I). Thus, the muscle pattern defects observed with both the gain-of-function and loss-of-function mutants are consistent with the argument that the gene *esg* participates in the regulation of adhesion processes, as previously proposed for *esg* function during tracheal system development [37], and that *sdc* is required for early Slit/Robo-signaling-dependent muscle guidance, as described recently (Figure 4E; [25]). Our results also show that *sdc*-dependent Slit signaling serves as a muscle attractant during a late phase of muscle guidance [19], since abnormal muscle elongations are observed in fully developed but unhatched *sdc* mutant larvae (Figure 4C and 4F).

### Conclusion

We identified a series of genes whose activity impairs muscle pattern formation when misexpressed in a defined pattern of epidermal cells that represent the migration substrate and/or the attachment sites for the outgrowing myotubes and muscle fibers. The 66 identified candidate genes were selected from an EP-element insertion library composed of more than 4,500 individual lines. This number suggests that about 1.5% of *D. melanogaster* genes can affect muscle pattern formation when expressed in cells that are contacted by myotubes or muscles. Although the screening system can certainly be regarded as artificial, it nevertheless identified genes such as *Toll*, *gut feeling,* and *sulfateless* that have been previously implicated in muscle pattern formation because the corresponding loss-of-function mutations cause variable muscle phenotypes [28,32,40]. In addition, it identified genes whose products are known to participate in cell migration and/or cell targeting processes in the embryo. These genes include *esg* and *sdc* [25,37], and, as shown in Figure 4, loss-of-function mutations in these two genes cause a defective muscle pattern in the embryo, indicating that the activity of these genes is essential for embryonic muscle development.

It is interesting to note that the misexpression screen identified, in addition to the HSPG Sdc, a number of other muscle-pattern-disturbing genes that code for factors known to participate in carbohydrate side chain synthesis or side chain modification. Thus, these enzymes are likely to participate in communication events between muscle and epidermal cells, processes that may also involve signaling molecules in addition to Slit [18,19]. Similarly, the independent identification of three components of the Ubiquitin system suggests that Ubiqitin modifications of proteins or their stability play a role in muscle pattern formation. The plethora of factors identified here open a new avenue towards a detailed functional analysis of processes underlying the interplay of myotubes, their epidermal migration substrate, and the specialized segmental border cells to which myofibers ultimately attach. In addition, they can be used towards developing an understanding of migratory processes in other developmental processes of *D. melanogaster* and, in view of the conservation of the genes identified here, possibly also in other species including mammals.

## Materials and Methods

### Genetics and expression detection.

To generate novel EP-element integration lines we used two different EP-elements. EPg was modified to function in the female germline and contains the *white^+^* gene as a selectable marker in *white* mutant individuals [23]. The second EP-element, P{Mae-UAS.6.11}, contains the *yellow* gene as a corresponding marker [42]. More than 8,500 independent EPg insertions were generated using a jump-starter line from an EPg insert on a CyO chromosome (EPg4–38), and 5,100 independent lines were established using P{Mae-UAS.6.11}. Chromosomes bearing an EP-element integration were kept either as homozygous lines or in *trans* to a corresponding balancer chromosome. EP-element-bearing males were crossed with *srmodGAL4*-bearing females, and their F1 offspring were screened for lethality. In case of lethality, candidates were crossed with *srmodGAL4-* and *srGAL4*-drivers and their F1 offspring were examined after staining with anti-MHC antiserum (kindly provided by D. Kiehart) using the staining protocol previously described [12]. Anti-sense DIG-labeled RNA probes were prepared and whole mount in situ hybridization was performed as described [12].

### Molecular analysis.

The EPg and the pMae elements have been previously described [23,42]. The *srGAL4* and the *srmodGAL4* lines were obtained by cloning the KpnI and XbaI of the *sr239* and *sr239*Δ*pan* DNA as described in [12] into the p221 vector (kindly provided by C. Klämbt). In order to determine the EP-element integration sites within genomic DNA, we performed inverse PCR as described on the Berkeley *Drosophila* Genome Project Web page (http://www.fruitfly.org/) with overnight digestion by either MaeI or Csp6I. Fragments were amplified for the 5′-end of P{EP,y+}; the primers used for the pMae were 5′-CAGCTGCGCTTGTTTATTTGC-3′ (forward) and 5′-TGGGAATTCGTTAACAGATCCAC-3′ (reverse), and for the EPg were Pw new up (CAG CCG AAT TAA TTC TAG TTC CAG TGA A) and Pw new low (ACT TCG GCA CGT GAA TTA ATT TTA CTC C). The amplified DNA was sequenced and used to determine the insertion site (see Table S1).

## Supporting Information

Table S1 Description of the Tested Insertion Lines The lines are ordered according to their names (Line). DG-EP denotes lines that carry the *P{Mae-UAS.6.11}*-element whereas HD-EP stands for lines that are generated by mobilization of the modified *EPg*-element. The chromosome arm (Arm), orientation (Strand), and position according to *D. melanogaster* genomic sequence release 3 (Position) are indicated. The 5′ sequence tag (forward strand) for each insertion line is listed (Sequence).
(607 KB XLS)

Table S2 Insertion Lines Available from the Bloomington Stock Center The lines are ordered according to the name under which they will be kept in the Bloomington Stock Center (Line). The former name as used in Table 1 (Old Name) is also listed. The chromosome arm (Arm) as well as the position according to *D. melanogaster* genomic sequence release 4.1 (Coordinate) is shown. The next gene (Gene) with its extension (5′ Gene and 3′ Gene, respectively) and orientation (Strand) as well as the relative position of the insert to the gene (Position) is indicated. The 5′ sequence tag for each insertion line is listed under Sequence.
(152 KB XLS)

Table S3 Identified Candidate Genes The candidate genes are arranged into groups by their proposed biological function. For each candidate, the CG number (according to the FlyBase [http://flybase.bio.indiana.edu/]), the gene synonym, the EP number, the orientation of the expressed transcript, predicted protein domains, the biological process, the expression pattern, the criteria for the validation of candidate genes, and a description of the gain-of-function muscle phenotype are listed. The wild-type expression patterns are based on the Berkeley *Drosophila* Genome Project in situ expression data [39], Berkeley *Drosophila* Genome Project CHIP-expression data, or in situ hybridization using either genomic fragments (gen frag) or Ests. In this case the name of the Est used is listed. Abbreviations used to describe the expression are as follows: Ap, muscle attachment sites; Br, brain; Ep, epidermis; Fb, fatbody; Gc, garland cells; Go, gonads; He, heart; Hg, hindgut; mat, maternal expression; Md, early mesoderm; Mg, midgut; Ml, ventral midline; Mu, muscles; Nb, neuroblasts; Pc, pericardial cells; Sb, epidermal segment border; Sg, salivary glands; Tp, tracheal placodes; Ts, tracheal system; zyg, zygotic expression. The criteria used to validate the identified candidate genes were (1) reversion of the EP-element, (2) induction of expression, (3) similar phenotype induced by an UAS cDNA transgene, (4) additional EP-element, and (5) published data. Anti-sense candidates were only considered in the absence of a gene in sense orientation within 10 kbp downstream of the EP-element (6).
(180 KB DOC)

## Abbreviations

*esg*: *escargot*
HSPG: heparan sulfate proteoglycan
MHC: Myosin heavy chain
Sdc: Syndecan
UAS: upstream activating sequence
