# Supplementary material for: Gain-of-Function Screen for Genes That Affect Drosophila Muscle Pattern Formation
Source: PLoS Genet. 2005 Oct 28;1(4):e55. doi: 10.1371/journal.pgen.0010055 (PMC1270011; doi:10.1371/journal.pgen.0010055)
Supplement: Table S3 — The candidate genes are arranged into groups by their proposed biological function. For each candidate, the CG number (according to the FlyBase [http://flybase.bio.indiana.edu/]), the gene synonym, the EP number, the orientation of the expressed transcript, predicted protein domains, the biological process, the expression pattern, the criteria for the validation of candidate genes, and a description of the gain-of-function muscle phenotype are listed. The wild-type expression patterns are based on the Berkeley Drosophila Genome Project in situ expression data [39], Berkeley Drosophila Genome Project CHIP-expression data, or in situ hybridization using either genomic fragments (gen frag) or Ests. In this case the name of the Est used is listed. Abbreviations used to describe the expression are as follows: Ap, muscle attachment sites; Br, brain; Ep, epidermis; Fb, fatbody; Gc, garland cells; Go, gonads; He, heart; Hg, hindgut; mat, maternal expression; Md, early mesoderm; Mg, midgut; Ml, ventral midline; Mu, muscles; Nb, neuroblasts; Pc, pericardial cells; Sb, epidermal segment border; Sg, salivary glands; Tp, tracheal placodes; Ts, tracheal system; zyg, zygotic expression. The criteria used to validate the identified candidate genes were (1) reversion of the EP-element, (2) induction of expression, (3) similar phenotype induced by an UAS cDNA transgene, (4) additional EP-element, and (5) published data. Anti-sense candidates were only considered in the absence of a gene in sense orientation within 10 kbp downstream of the EP-element (6). (180 KB DOC) [file pgen.0010055.st003.doc]

| **CG number** | **gene** | **EP-line** | **Orien.** | **Functional domains** | **Biological process** | **Expression** | **Criteria** | **Gain-of-function**  **Muscle phenotype** |
| --- | --- | --- | --- | --- | --- | --- | --- | --- |
| **Secreted and membrane associated factors** | | | | | | |  |  |
| CG14052 |  | EP(1)1460 | sense | sig. peptide | unknown | gen frag: mat. zyg weak ubiq. | (3, 5) | medium |
| CG6301 |  | EP(2)2269 | anti | sig. peptide | unknown | BDGP: no data | (5, 6) | extreme, germb. retr. abn. |
| CG17368 |  | EP(3)3024 | sense | sig. peptide | unknown | BDGP: no data | (5) | strong |
| CG33148 |  | EP(2)2470 | sense | Ca2+ and phospho lipid bdg. | unknown | BDGP: no data | (5) | var. musc. mis. & ext. |
| CG31475 |  | EP(3)3186 | sense | TM + 4EF-hand motifes | unknown | BDGP: no data CHIP: expr. entire embr. | (5) | str.: musc. mis |
| CG9030 |  | 10906 | anti | 3 TM domains | unknown | BDGP: bad stain. Sb | (6) | var. extreme |
| CG10106 | *tsp42Ee* | 20290 | anti | Tetraspanin | unknown | BDGP: mat.,SB, Ml, weak ubiq. | (6) | medium |
| CG10497 | *sdc* | 21140 | sense | HSPG | slit/robo signalling | mat., TP, SB, Ap | (1, 2, 3) | var. musc. missing |

| CG30023 | *sprt* | 25604 | sense | PDZ domain prot. | unknown | BDGP: Md, heart, Epi stripes | (2) | var. weak to strong |
| --- | --- | --- | --- | --- | --- | --- | --- | --- |

| CG31349 | *pyd* | 35117 | anti | Guanylat Kinase (MAGUK) | jnk signalling | BDGP: mat., Md, Sb, Hg, Ts, Ml, Ap | (6) | strong, few extreme |
| --- | --- | --- | --- | --- | --- | --- | --- | --- |
| CG33207 | *pxb* | 35134 | sense | TM domain | smo signalling | SD26190: wg-like, Mg, part. Ep stripes | (2) | medium, few extreme |
| CG14713/  CG14714 fus. |  | 35141 | sense | tyrosin phosphatase | unknown | AT17253: mat,Ml, part. Ep stripes, Gc | (2) | weak few medium |
| CG5490 | *toll* | 36772 | sense | receptor | dorsal signalling | mat, dor Ep, Ep stripes, Ts, Sb, part. Ap | (2, 3) | weak |
| **Cytoskeleton** | | | | | | |  |  |
| CG9910 | *kat80* | EP(1)1319 | sense | microtubule bdg. | cytoskeleton org. | BDGP: mat. / zyg. unclear | (4, 5) | mild |
| CG9553 | *chic* | 25871 | sense | PIP(4,5)-P2 bdg. | cytoskeleton org. | BDGP: mat, CNS | (4) | var. weak to ext. |
| CG13913 |  | 35048 | sense | pot. actin bdg. (FH3-dom.) | unknown | RE53394: dor. + ven Ep, Sb | (2) | weak at 29°C |
| **Transcription factors and RNA binding proteins** | | | | | | |  |  |
| CG4059 | *ftz-f1* | EP(3)0447 | anti | Transcription factor | gene expression | mat. | (5) | str., elon. muscles, detach. |
| CG12701 |  | EP(1)1344 | sense | pot. ZnF-Transcription factor | unknown | LD47819: mat, CNS, Br | (2, 3, 5) | strong, muscle exten. |
| CG3758 | *esg* | EP(2)2159 | sense | transcription factor | gene expression | Ep,Tp,PNS,CNS,Ml,Discs | (5) | mild, & var. |
| CG1414 | *bbx* | 10912 | anti | HMG-transcription factor | unknown | BDGP: str. mat, Mg, Hg, Sg | (6) | strong & var. |

| CG8920 |  | 25585 | sense | Tudor motif ssDNA or RNA bdg. | unknown | BDGP: mat, Mg, Mu | (2) | var.: weak to extreme |
| --- | --- | --- | --- | --- | --- | --- | --- | --- |

| CG7734 | *shn* | 25866 | sense | ZnF-Transcription factor | gene expression | dors. EP, Md, gut, Ts | (4) | str. musc. missing |
| --- | --- | --- | --- | --- | --- | --- | --- | --- |
| **Protein modification and degradation** | | | | | | |  |  |
| CG32629/  CG32632 fus. |  | EP(1)1218 | sense | Tyrosyl sulfotransferase2 | protein modifi. | AT12436: mat, Sg, Ap, Hg, Mg, Ep, Pc | (5) | mild |
| CG31973 |  | EP(2)2220 | sense | sig. Peptide, chitin bdg., transferase | extracellular carbohydrate modif. | BDGP: no data | (5) | strong: mus. miss. |
| CG2864 | *parg* | 10914 | sense | poly(ADP-ribose) glycohydrolase | carbohydrate metabolism | gen frag.: mat, Md, Mg | (2) | var. mus. miss. |
| CG5008 | *gnbp3* | 32189 37027 | sense | glucosidase activity | extracellular carbohydrate modifi. | SD21560: single Ep cells, parts of Sb | (2, 4) | medium |
| CG8339 | *sfl* | 35161 | anti | heparan sulfate-glucosamine-N-sulfotransferase | heparan sulfate modification | mat, weak ubiq. | (6) | extreme |
| CG11033 |  | 3093 | sense | F-Box | protein degradation | SD04170: mat, Md, Mg, weak ubiq. | (1, 5) | variable |
| CG1782 | *uba1* | 25812 | anti | ubiquitin ligase | protein degradation | BDGP: mat, weak ubiq., Br, CNS | (4) | variable |
| CG7425 | *ubc/eff* | 32278 | sense | ubiquitin conjugating enzyme | protein degradation | GH14739: mat, ubiq. | (2) | medium (extreme at 29°C) |
| **Cell cycle control** | | | | | | |  |  |
| CG4965 | *twe* | EP(2)0613 | sense | protein tyrosine phophatase | cell cycle | mat. / in adult: testis | (5) | variable |
| CG9999 | *sd* | EP(2)1173 | sense | Ran GTPase activator | cell cycle | mat., Nb, Br, Go | (5) | variable |
| CG5814 | *cycB3* | EP(3)3127 | sense | kinase activator subunit | cell cycle | mat, Nb, | (5) | strong: mus. missing |
| CG5940 | *cycA* | 30838 | sense | kinase activator subunit | cell cycle | mat, CNS, PNS | n. det. | strong |
| **Biosynthesis** | | | | | | |  |  |
| CG6510 | *rpL18A* | EP(2)2281 | sense | ribosomal protein | translation | BDGP: no data | (5) | variable, weak to extr. |
| CG9742 |  | 10913 | sense | snRNP | splicing | BDGP: early ubiq., late gut | (2) | weak, abn. mus. attach. |

| CG5442 | *sc35* | 20080 | sense | SR splicing | splicing | BDGP: mat., Md, weak Mg. | (2) | weak & var. |
| --- | --- | --- | --- | --- | --- | --- | --- | --- |

| CG2163 | *pabp2* | 21143 | sense | mRNA polyadenylation bdg. | mRNA processing | BDGP: mat., ubiq. | (2, 4) | var. extreme |
| --- | --- | --- | --- | --- | --- | --- | --- | --- |
| CG10685 |  | 26450 | sense | RNA pol subunit | transcription | BDGP: no data | n. det. | strong |
| CG10685 | *RNApolII* | 32222 | sense | RNA transcription | transcription | BDGP: no data | n. det. | medium |
| CG10851 | *B52* | 35047 | sense | SR-splicing factor | splicing | LD37428: mat, Br, CNS, Md | (2) | var.: medium to extreme |
| **CG number** | **gene** | **Line** | **Orien.** | **Functional domains** | **Biological process** | **Expression** |  |  |
| **Enzymatic activities** | | | | | | |  |  |
| CG8938 | *gstS1* | EP(2)1074 | sense | glutathione transferase act. | oxidative stress, cytoskeleton org. | mat.; Md, Hg, Pv, Fb, dor. Ep | (4) | variable |
| CG16747 | *oda* | EP(2)2104 | sense | enzyme inhibitor act. | cell differentiation | mat. Mg, weak ubiq. | (5) | str. musc. missing |
| CG9078 | *ifc* | EP(2)2092  20692 | sense | sphingolipid delta-4 desaturase | lipid modification | mat, Pc, Mg, Md | (4, 5) | weak |
| CG17292 |  | EP(2)2392 | sense | Triacylglyceryl-Lipase | homeostasis | BDGP: no data | (5) | str. musc. missing |
| CG17146 | *adk1* | 32155 | sense | adenylate kinase act. | ATP metabolism | BDGP: no data | n. det. | var.: weak to strong |
| CG12078 |  | 35064 | sense | pot. Phosphatase | unknown | BDGP: no data | (4). | weak, few musc. mis. or not attached |
| CG6854 |  | 35098 | sense | CTP-Synthase | pyrimidine base metabolism | BDGP: no data | n. det. | extreme at 29°C |
| CG7858 | *mocs1* | 35124 | sense | synthesis of molybdenum cofactor | unknown | BDGP: no data | (4) | strong |
| CG31549 |  | 35180 | sense | oxidoreductase act. | lipid metabolism | BDGP: maternal, weak midgut | (2, 4) | strong |
| CG9287 |  | 36698 | anti | carboxylic ester hydrolase | unknown | BDGP: no data, CHIP: embr. expr. | (6) | variable |
| **Transporter and Carrier proteins** | | | | | | |  |  |
| CG17818 | *rdgB-β* | EP(2)2360 | sense | Phosphatidylinositol transfer prot | unknown | LD14189: mat., Md, Mg, weak ubiq. | (5) | str,: musc. miss. & exten. |
| CG11665 |  | EP(2)2646 | sense | monocarboxyl acid transporter | pot. cation transport | BDGP: no data | (5) | weak & var. |

| CG10444 |  | 25650 | sense | Na-dep. transporter | unknown | BDGP: maternal, Chip weak zygot. | (2) | var: weak to extreme |
| --- | --- | --- | --- | --- | --- | --- | --- | --- |

| CG11857 |  | 32235 | sense | has RER1 domain | intracellular vesicle tr. | LP10429:mat, Sg, weak ubiq. | (2) | R.T. none/29°C strong |
| --- | --- | --- | --- | --- | --- | --- | --- | --- |
| CG4963 |  | 35059 35145 | sense | mitochondrial carrier | transporter | GH09840: str. Mg, weak ubiq. | (2, 4) | medium, few musc. mis. |
| **Factors with unknown function** | | | | | | |  |  |
| CG8441 |  | EP(2)0969 | sense |  | unknown | BDGP: no data | (4, 5)) | Lt musc. mis. & abn. posi. |
| CG31710 |  | EP(2)2160 | sense |  | unknown | gen.Frag: weak ubiq. | (5) | str.: musc. mis. & long ext. |
| CG12842 |  | EP(2)2425 | anti |  | unknown | BDGP: no data | (5) | weak, unclear |
| CG12996 |  | 10919 | sense |  | unknown | gen frag.: mat., Md, weak Mg | (2) | strong, head invol. def. |
| CG17059 |  | 26140 | sense |  | unknown | BDGP: no data CHIP: emb. expr. | (2) | var: str. to extreme |
| CG12128 |  | 26462 | sense |  | unknown | LD07909: mat., Md, Mg, weak ubiq. | (2) | var.: weak to extr. (at 29°C) |
| CG5478 |  | 32272 | sense |  | unknown | AT06251:mat, ubiq. | (1, 2, 3) | var. medium to extreme |
| CG32436 |  | 35012 | sense |  | unknown | BDGP: no data | (2) | strong |
| CG3563 |  | 35062 | sense |  | unknown | LD15689:mat, post. Mg, Sb | (2) | weak |
| CG14648 |  | 36553 | sense |  | unknown | LD30155: mat, Md, Mg, weak ubiq. | (1, 2, 3) | strong , mus. miss. & ext. |
